# Effect of Conducting Polyaniline/Graphene Nanosheet Content on the Corrosion Behavior of Zinc-Rich Epoxy Primers in 3.5% NaCl Solution

**DOI:** 10.3390/polym11050850

**Published:** 2019-05-10

**Authors:** Yanhua Lei, Zhichao Qiu, Jiurong Liu, Dongdong Li, Ning Tan, Tao Liu, Yuliang Zhang, Xueting Chang, Yanhong Gu, Yansheng Yin

**Affiliations:** 1 Institute of Marine Materials Science and Engineering, College of Ocean Science and Engineering, Shanghai Maritime University, Shanghai 201306, China; qzc4321@163.com (Z.Q.); liujingrong@126.com (J.L.); 201730410071@stu.shmtu.edu.cn (D.L.); tanning986@163.com (N.T.); liutao@shmtu.edu.cn (T.L.); ylzhang@shmtu.edu.cn (Y.Z.); xtchang@shmtu.edu.cn (X.C.); ysyin@shmtu.edu.cn (Y.Y.); 2 Beijing Key Laboratory of Pipeline Critical Technology and Equipment for Deepwater Oil & Gas Development, Beijing Institute of Petrochemical Technology, Beijing 102617, China

**Keywords:** conducting polymer, PANI, LEIS, graphene, corrosion

## Abstract

The corrosion behavior of zinc-rich epoxy primers or paints (ZRPs) with different conducting polyaniline-grafted graphene (PANI/Gr) contents was investigated. Conductivity of the formed PANI/Gr nanosheets was significantly improved by employing the Gr as the inner template to synthesize the PANI. The protective properties and electrochemical behavior of coatings with artificial defects were investigated by monitoring the free corrosion potential versus time and by using localized electrochemical impedance spectroscopy (LEIS). A synergetic enhancement of the physical barrier role of the coating and the zinc sacrificial cathodic protection was achieved in the case of ZRP including PANI/Gr nanosheets. In addition, the ZRP mixed with the PANI/Gr at a content of 0.6% exhibited the best anticorrosion performance across the range of investigated PANI/Gr contents.

## 1. Introduction

Organic coatings are applied to protect steel from corrosion due to their inhibition behavior, barrier function, and cathodic protection [1]. Zinc-rich epoxy primers or paints (ZRPs) are widely used as cathodic protection in a variety of severe environments via two protective mechanisms. First, sufficient electrical contact between coating and substrate is established because of the high zinc content. After the electrolyte diffuses into the coating, the zinc particles provide cathodic protection via the sacrificial anodic dissolution, thereby promoting the electrochemical process of a Zn–Fe microcell. Second, the zinc corrosion products (i.e., zinc oxide, zinc hydroxide, and hydrozincite) fill the coat pores and may also act as an electrical insulator under normal conditions [2]. As the organic and inorganic binders which are commonly used in ZRPs are naturally non-conductive, the electrical contact between the spherical zinc particles and steel substrates could not be maintained for a long time. Therefore, high zinc content (normally higher than 65 wt %) is necessary to ensure electrical conductivity [3]. Nevertheless, the utilization ratio of the zinc particles is very low [4], which is a waste of resources and results in environmental pollution.

Numerous efforts have been made to prolong the ZRP’s lifetime through two routes. One way is to improve the barrier effect of the ZRP with graphene oxide (GO) [5], nano clay [6], TiO_2_ [7], and so on. The addition of these fillers could extend the diffusion path of the electrolyte, which can reduce the rate of zinc particle consumption. However, the utilization ratio is still low. The other way is to improve the conductive property of the coatings by introducing different types of electrical conducting additives, for example, grephene (Gr) [8], polypyrrole (PPy) [4], polyaniline (PANI) [9,10], carbon nanotube (CNT) [11], carbon black [12], Al [13,14,15], and Co [16]. The improved conductive property could make it easier to link zinc particles and the steel substrate. In this way, the utilization ratio of zinc particles is increased while good conductivity often results in a decreased barrier effect due to the easy charge transfer.

Hayatdavoudi and Rahsepar [8] applied Gr to modify a ZRP coating, and pointed out that the presence of Gr nanosheets in the ZRP coating could provide not only an improvement in electrical contact but also an enhanced barrier against aggressive species. The impedance value was very low, almost under 1 kΩ∙cm^2^, which may be attributed to the superior conductivity of the coating by adding the good conductive Gr. Interestingly, Ramezanzadeh et al. [5] employed highly crystalline and conductive PANI/GO composites as an additional filler into a ZRE coating. They revealed that GO acted as a barrier and PANI enhanced the conductive behavior, which resulted in a higher cathodic protection duration. We compared the effects of Gr, PANI, and polyaniline-grafted graphene (PANI/Gr) on the corrosion performance of ZRPs [17], and considered that the combined PANI/Gr was a more effective strategy to enhance the protection performance of ZRP coatings compared to Gr. However, the quantity of added PANI/Gr nanosheets must be optimized to enhance the protection performance of ZRP coatings. Thus, in this work, the corrosion behavior of ZRP samples was studied with different PANI/Gr contents in the range of 0–0.8 wt %. The influence of PANI/Gr content on the corrosion protection behavior was evaluated in 3.5% NaCl solution by electrochemical impedance spectroscopy (EIS) and by monitoring the free corrosion potential versus time. Localized electrochemical impedance spectroscopy (LEIS), scanning electron microscopy (SEM) coupled with energy-dispersive X-ray spectroscopy (EDS), and X-ray photoelectron spectroscopy (XPS) were also utilized to support the electrochemical mechanism findings.

## 2. Materials and Methods

Gr nanosheets with thicknesses of 1–5 nm and widths of several microns were obtained from XianFeng Nano Co. (Suzhou, China). The conductivity of the purchased Gr nanosheets was in the range of 1200–1500 S cm^−1^. Aniline monomer (Ani) with purity of 99% purchased from Aladdin Co. (Shanghai, China) was used without any pretreatment. All the other chemicals were of analytical grade and were used without further purification. The solutions were prepared using double-distilled and subsequently deionized Millipore water. DH32 steel obtained from Baowu Steel Co. (Shanghai, China) was used as the substrate. Specimens for the corrosion measurements were cut from the DH32 steel to a size of 10 × 10 mm. Prior to the experiment, the specimen surface was ground to an 800-grit SiC paper, then degreased ultrasonically with acetone and ethane, and washed with triply distilled water. Following this pretreatment, the steel as work electrode was immediately transferred to the electrochemical cell.

**Figure 1:**
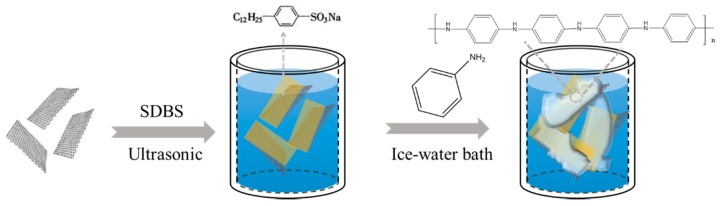
The synthesis procedures of polyaniline-grafted graphene (PANI/Gr).

Synthesis of PANI/Gr: PANI/Gr composites were prepared by in situ chemical polymerization of Ani on Gr. In a typical synthesis, a 100 mg portion of Gr was first dispersed in 200 mL 0.1 M sodium dodecyl benzene sulfonate (SDBS) by ultrasonic treatment for 2 h under ambient conditions to give a dark dispersion. Then, the suspension was cooled to 2 °C in an ice-water bath. Ani monomer with a volume of 1 mL was dissolved in 20 mL 0.2 M HCl and the mixture was injected into the previous suspension with an injection springe, then further dispersed for 1 h with strong stirring. Afterwards, an equal volume of a 0.25 M ammonium persulfate (APS) solution was slowly added dropwise into the above mixture and kept at 0–4 °C for 24 h with strong stirring. Finally, the PANI/Gr composites were washed with distilled water and ethanol, dried in a vacuum oven at 60 °C for 24 h. The weight ratio was evaluated by comparing the product weight with the presence and absence of Gr during the PANI synthesis procedure under the same conditions. The synthesis procedures are schematically presented in Figure 1.

### 2.1. Characterization of the Functional Nanoparticle or Composites

The synthesized PANI powder and PANI/Gr were characterized by SEM, TEM, Fourier-transform infrared spectroscopy (FT-IR), and Raman spectroscopy (RS). The surface morphologies and compositions were characterized using a SEM-EDS, JSM-6510LA (JEOL, Tokyo, Japan). The TEM observation were carried out on a JEM-2100F (JEOL, Tokyo, Japan). The formed PANI layer was analyzed by RS and FT-IR. For RS, a Raman polychromator spectrometer (Bunko-Keiki M30-TP-M, Tokyo, Japan) was used with excitation by YVO_4_ solid-state laser at 532.0 nm wavelength. The laser power for the excitation was controlled below 5 mW to avoid damage to the PANI films. FT-IR spectra were measured in the wavenumber range of 4000–400 cm^−1^ at a resolution of 4.0 cm^−1^ by using Vertex-70 (Bruker, Ettlingen, Germany). XPS measurements were performed on a JPS-9200 (JEOL, Tokyo, Japan) with a Mg Ka (1253.6 eV) radiation. The calibrations of the binding energies were referenced to the Au 4f7/2 electron peak at 84.00A eV. The DC electrical conductivity of the powder was measured using a multifunction digital four-probe electrical conductivity measuring instrument (ST4742B, Suzhou Jingge Electronic Co. Ltd., Jiangsu, China). The AC dielectric properties of the ZRP coatings treated with different pigments were determined using an impedance analyzer Agilent E4980AL (Agilent, Santa Clara, CA, USA).

### 2.2. Preparations of Coated Specimens

A commercially available ZRP (provided by Sanmu Group Co., Jiangsu, China) was used to prepare various coating compositions. The ZRP containing 80 wt % zinc particles was composed of an epoxy resin (E44) and a polyamide hardener (T31) with the weight ratio of 4:1. To prepare the PANI/Gr-modified ZRP (PANI/Gr-ZRP) coating, different weight percentages were dissolved in dimethylbenzene by ultrasonic stir for 30 min and then added into the epoxy resin containing 80 wt % zinc. After stirring at room temperature until volatilization of dimethylbenzene finished, the resin was coated onto the DH32 steel. The samples were exposed to air for one week to ensure the absolute solidification of the resin. The ZRP formulation containing PANI/Gr is numbered and given in Table 1. Thickness of the coatings was determined using a PosiTector6000FNS2 probe (DeFelsko Co. Ltd., Ogdensburg, NY, USA), a coating thickness measuring instrument.

### 2.3. Corrosion Test of the Modified ZRPs

The protection properties of the PANI/Gr on the DH32 steel were studied in a solution containing Cl^−^ at 25.0 ± 0.3 °C under atmospheric conditions, in which the open circuit potential (OCP) and EIS were measured. In order to study the effect of the PANI/Gr nanocomposite segments on the cathodic sacrificial behavior of the ZRPs, the coated samples were first immersed into a 3.5 wt % NaCl solution for 24 h. Then, an artificial defect with a size of 10 × 0.2 mm (which means the defect penetrated the coating) was induced for each coated sample. The samples with artificial defect were then immersed into the NaCl solution, and the OCP was monitored during the whole period of immersion.

Electrochemical impedance spectroscopy (Autolab, Metrohm, Zurich, Switzerland) was used to evaluate the protection capacity of the steel sheets coated with ZRP and PANI/Gr-ZRP. A conventional three-electrode electrochemical cell, composed of platinum as counter electrode, a saturated calomel electrode (SCE) as reference, and the coated steel panel as working electrode, was used to carry out the electrochemical tests in 3.5 wt % NaCl solution. The test was done at OCP in the frequency range and amplitude sinusoidal voltage of 10^−2^ Hz to 10^5^ Hz (peak to zero) and 20 mV, respectively. The software program ZSimpwin (E Chem Software, Ann Arbor, MI, USA) were used to fit the impedance data.

Localized electrochemical impedance spectroscopy (LEIS) of the coatings in the presence of an artificial defect of 2 × 0.2 mm was performed on an M470 scanning electrochemical workstation (Bio-Logic, Paris, France). A three-electrode cell was used to perform the LEIS measurement. An SCE and a carbon rod were used as the reference electrode and the counter electrode, respectively. The coated sample was studied as the working electrode. During LEIS testing, a distance of 100 μm was adjusted from the electrode surface to a microprobe Pt tip with a tip diameter of 5 μm. A current signal with amplitude of 10 μA at a single frequency of 10 Hz was generated by the electrochemical workstation to finish the LEIS measurements. All the potentials in this paper were plotted with respect to the Ag/AgCl/sat. KCl (0.197 V vs. Standard hydrogen electrode(SHE)). The sacrificial protection performance of the samples with X-cut scribes was investigated in a 5 wt % NaCl solution at 45 °C by salt spray test in a salt spray cabin KLT-90 (China Electronics Technology Group Co., Beijing, China).

## 3. Results

### 3.1. Characterization of the PANI/Gr Composites

**Figure 2:**
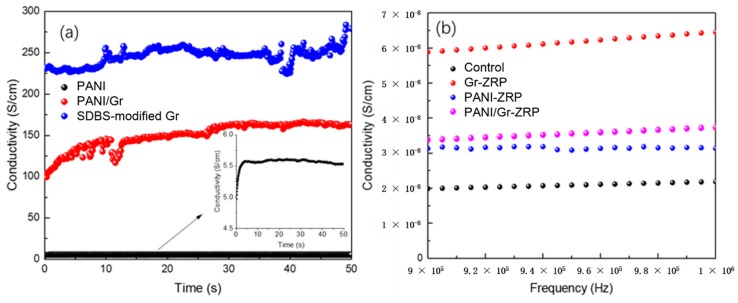
(**a**) The DC conductivity results of the synthesized PANI, the PANI/Gr nanocomposites, and the sodium dodecyl benzene sulfonate (SDBS)-modified Gr; (**b**) the AC conductivity results of the ZRP coatings with the addition of different conductive pigments.

Since the conductivity of additions would significantly influence the corrosion performance of the ZRP primer, the conductivity of the addition was therefore first measured. Figure 2a shows the conductivities of the synthesized PANI particles and PANI/Gr nanosheets. The conductivity of the PANI sample was evaluated at 5 S cm^−1^, which was comparable to the results of Stejskal and Gilbert [18] and Meroufel et al. [19], indicating the formation of the emeraldine salt (PANI-ES) state. However, the conductivity after introducing the Gr during the in situ synthesis process increased to 150 S cm^−1^, which is more than one order of magnitude. It must be noted that although the modification of Gr with SDBS decreased the conductivity of the Gr, the conductivity of the pretreated Gr was still much higher than that of the synthesized PANI/Gr composites. The results of the DC conductivity measurement indicated that the PANI was successfully synthesized on the surface of Gr, and the formation of PANI on the surface of Gr reduced the conductivity of the Gr. During the synthesis procedure, when Ani monomers were added into the Gr suspension, the Ani monomers (being the electron donor) could immediately absorb onto the surfaces of Gr (being the electron acceptor) and form a type of weak charge transfer complex due to the electrostatic attraction [20]. The weight ratio of the PANI to Gr in the synthesized PANI-Gr composites was analyzed by comparing the product weight with the presence and absence of Gr during the PANI synthesis procedure under the same conditions, the results of which indicated a ratio of nearly 2:1. Then, the AC conductivity of the ZRP coatings treated with the above pigments was evaluated. As shown in Figure 2b, compared to the ZRP coating, the AC conductivity of the ZRP coatings was improved to different degrees after the dispersion of the PANI, PANI/Gr, and Gr conductive pigments. Therefore, considering the excellent barrier property of PANI, it was assumed herein that the inclusion of PANI/Gr nanocomposites with a reasonable content in ZRP would obtain a better synergistic protection of the barrier and cathodic protection of the ZRP coating.

**Figure 3:**
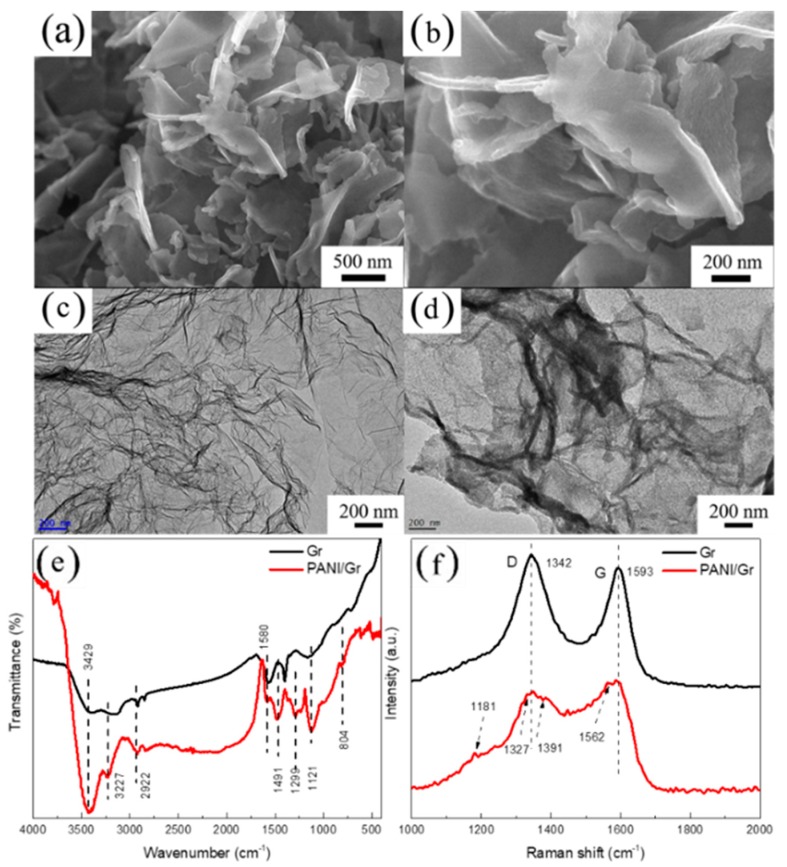
(**a**) SEM image of the synthesized PANI/Gr; (**b**) High magnification of the SEM image; (**c**) TEM image of graphene; (**d**) TEM image of the synthesized PANI/Gr; (**e**) Comparison FT-IR spectra; (**f**) Raman spectra of the modified Gr and PANI/Gr.

The characterization of the synthesized PANI/Gr composites was carried out by SEM, TEM, FT-IR, and Raman, as shown in Figure 3. The SEM images reveal that the synthesized PANI/Gr nanocomposites consisted of nanosheets with a thickness of nearly 10–20 nm, which is thicker than the Gr nanosheets. A high-resolution SEM image is given in Figure 3b, in which PANI nanosheets consisting of small nanodots are observed. In the synthesis process, the aniline monomer was initially adsorbed onto the surface of the graphene nanosheets due to electrostatic attractions, and then in situ polymerization took place. From the TEM observation, the typical wrinkled structure of Gr remained after PANI grafting, as demonstrated in Figure 3c,d. The formation of PANI on Gr was characterized with FT-IR and Raman spectroscopy. Figure 3e compares the FT-IR spectroscopy of pristine Gr with the PANI/Gr nanocomposites. The stretching modes of C–C and C=C in the Gr occur at the absorptions of 1402 and 1580 cm^−1^, respectively. The presence of the surface functional groups on graphene after PANI grafting was further confirmed by FT-IR. The O–H and N–H stretching modes give rise to the strong broad band at 3429 cm^−1^ and weak band at 3227 cm^−1^, respectively. The C–H stretching band of the aromatic ring occurs at 2922 cm^−1^, and the absorption peak intensity increased obviously due to the PANI formation to the surface of Gr. Other characteristic peaks related to the PANI structure can also be seen at 1580 cm^−1^, 1491 cm^−1^, and 1299 cm^−1^, which are assigned to C–C=C conjugative backbone stretching, C=N stretching, and C–N stretching vibration with aromatic conjugation, respectively [21,22,23,24]. The peak at 1121 cm^−1^ corresponds to the in-plane bending vibration of C–H [25], whereas the peak at 804 cm^−1^ corresponds to the out-of-plane bending vibration of C–H. All the above peaks can be distinguished from the spectrum of PANI/Gr composites, which indicates the successful formation of PANI on the surface of graphene. Figure 3f shows the Raman spectra for the synthesized PANI/Gr nanocomposite and graphene. The G and D peaks of Gr are recorded at 1580 and 1350 cm^−1^, arising from the stretching of the C–C bond and the disorder in the sp2-hydridized Gr structure, respectively [26]. For the neat Gr, it is observed that the *I*_D_/*I*_G_ is quite high, which indicates that the Gr used is rich in defects. After the polymerization of PANI on Gr, it is evident that the *I*_D_ intensity was enhanced due to the sp2 C in the aromatic ring. Thus, the *I*_D_/*I*_G_ of the PANI-Gr decreased compared to that of the neat Gr. In addition, the PANI/Gr composite presents a weak shift of the D peak towards low wavenumbers resulting from the π–π* electron interaction between Gr and the Ani monomer [20]. Besides the wavenumber shift of the G and D peaks, the characteristic vibrational peaks of PANI are also evident in the PANI-Gr nanocomposite spectrum. The 1562 cm^−1^, 1372 cm^−1^, and 1189 cm^−1^ peaks can be attributed to the bipolaronic N–H bending vibration, the C–N stretching vibration of the cation radical species, and the C–H bending of the quinoid ring [27], respectively. All of the above results definitely indicate the formation of PANI on the surface of Gr.

### 3.2. OCP Measurements

**Figure 4:**
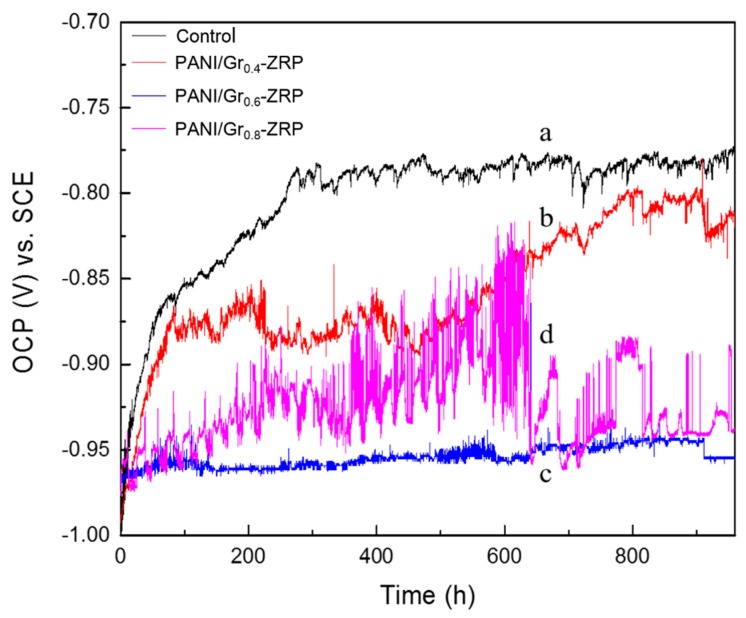
Open circuit potential (OCP) in 3.5% NaCl solution of DH32 steel coated with ZRP (control) (**line a**), PANI/Gr_0.4_-ZRP (**line b**), PANI/Gr_0.6_-ZRP (**line c**), and PANI/Gr_0.8_-ZRP (**line d**).

It is generally accepted that the OCP criterion for cathodic protection is lower than −0.76 V vs. SCE [28]. Thus, it is considered that the cathodic protection ability of substrate will be maintained if the corrosion potential of the coated substrate is kept below −0.8 V vs. SCE. OCP was monitored continuously during 1000 h of immersion. The OCP changes in the ZRP with different contents of PANI/Gr during immersion for up to 1000 h are plotted in line a in Figure 4. It was observed that the OCP of the control ZRP coating continuously increased with the extension of immersion time, and reached −0.76 V after 1000 h. All potentials of the ZRP coatings containing PANI/Gr remained lower than −0.8 V for up to 1000 h of immersion, which indicates that the substrates were protected cathodically. For example, the OCP of the PANI/Gr_0.4_-ZRP was raised to −0.84 V after 1000 h of immersion, although the OCP slowly shifted anodically with prolonged immersion, indicating the degradation of cathodic protection. The potential of both the PANI/Gr_0.6_-ZRP and PANI/Gr_0.8_-ZRP coatings maintained a low value during the whole period of immersion, although the potential of PANI/Gr_0.8_-ZRP fluctuated. Oxidation of the Zn to ZnO or other corrosion products due to the reduction of oxygen or PANI caused the fluctuation in the potential and the increase in the potential. Compared to the control samples, the doping of PANI/Gr extended the ZRP coating cathodic protection duration. The OCP of the PANI/Gr_0.6_-ZRP coating exhibited a more stable and negative potential during the long immersion test, which indicated that the ZRP coating with PANI/Gr_0.6_ could provide a better effective cathodic protection than the others.

**Figure 5:**
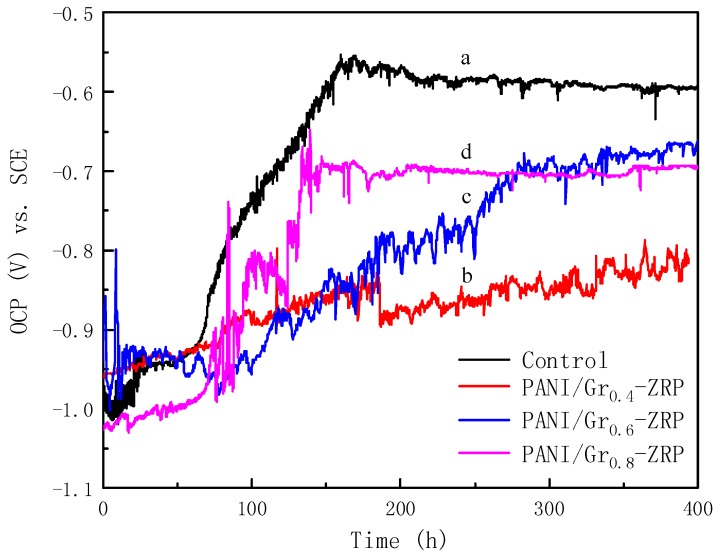
Open circuit potential (OCP) changes in 3.5% NaCl solution of samples with artificial defects: (**line a**) PANI/Gr_0_-ZRPs (control), (**line b**) PANI/Gr_0.4_-ZRP, (**line c**) PANI/Gr_0.6_-ZRP, and (**line d**) PANI/Gr_0.8_-ZRP.

In order to clearly study the content effects of PANI/Gr in the ZRPs on the cathodic sacrificial behavior, the changes in potential of the samples with artificial defects were also monitored as a function of immersion time. The ZRP coating with an artificial defect of 10 × 0.2 mm was immersed into the 3.5 wt % NaCl solution, and then the OCP was monitored throughout the whole process. Figure 5 presents the evolution of OCP for ZRPs with different contents of PANI/Gr and with immersion time. As can be seen in Figure 5, all potentials of the different coatings are initially in the range of −0.9 to −1.0 V, indicating the protection due to the cathodic scarification. For the control sample, the potential, initially at −1.0 V, shifts toward the positive direction with immersion, and reaches −0.6 V after 120 h of immersion. A decrease in the electroactive zinc area resulted in an increase in the potential, predicting a decrease in cathodic protection intensity. The introduction of defects to the coating accelerated the degradation of ZRP coatings’ cathodic protection, and the quick shift of potential can be attributed to the oxidation of Zn. However, the addition of electrically enhanced PANI/Gr segments led to the slight changes in potential. The OCP of the ZRP containing 0.4 wt % PANI/Gr rapidly increased with the extension of immersion, whereas for the 0.6 wt % PANI/Gr coating, the potential first fluctuated in the range of −0.8 to −1.0 V, then slowly increased to −0.76 V after 200 h of immersion. When the content of PANI/Gr increased to 0.8 wt %, the OCP values shifted to the positive region rapidly after 120 h and stabilized at −0.7 V during further immersion. It should be noted that the positive potential shift for the PANI/Gr-added coatings results mainly from the reaction couples of zinc oxidation and PANI reduction. Furthermore, the corrosion potential values of the PANI/Gr-ZRP at different contents are always lower than that of the control sample, as shown in Figure 5. This further indicates that some of the zinc particles were sacrificially active, thus contributing to the cathodic protection of the substrate with the artificial defects.

### 3.3. LEIS Measurement of the PANI/Gr-ZRP Coatings with Artificial Defect

**Figure 6:**
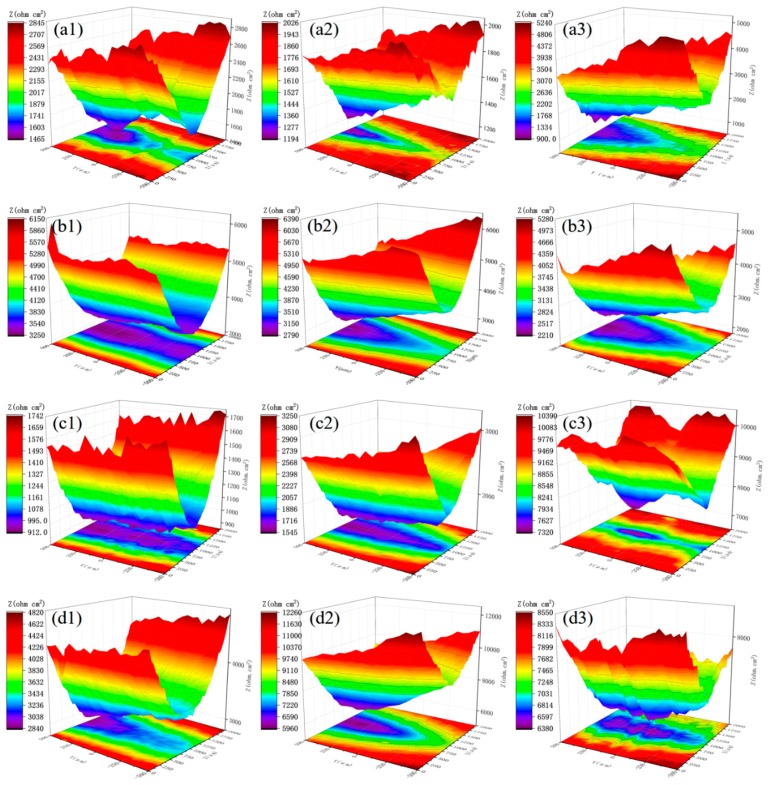
Localized electrochemical impedance spectroscopy (LEIS) results of the defect region for (**a**) PANI/Gr_0_-ZRPs, (**b**) PANI/Gr_0.4_-ZRPs, (**c**) PANI/Gr_0.6_-ZRPs, and (**d**) PANI/Gr_0.8_-ZRPs with artificial scratches immersed in 0.005 M NaCl solution for 48 h. (**a1**, **b1**, **c1,** and **d1**) are LEIS spectra for 1 h, (**a2**, **b2**, **c2,** and **d2**) are LEIS spectra for 24 h, and (**a3**, **b3**, **c3,** and **d3**) are LEIS spectra for 48 h.

To further study the effect of PANI/Gr addition on the coating cathodic protection, localized electrochemical impedance spectroscopy measurements were performed on a scratch-coated steel specimen with artificial damage which was exposed to a 0.005 M NaCl solution. Figure 6(a1–a3) shows the LEIS spectra for PANI/Gr_0_-ZRPs at different periods of immersion time, in which the local impedance in the scratched area remained at a low value during the exposure of 48 h. Additionally, it can be seen that the section of the scratched area with the low impedance value was enlarged after 48 h. The local impedance value in the scratched area of the PANI/Gr_0.4_-ZRPs was slightly higher than the PANI/Gr_0_-ZRPs at the beginning of immersion, approaching Figure 6b, and with the immersion time extension, the local impedance values initially increased lightly and decreased at 48 h, whereas the lowest impedance value barely changed during immersion. With the content of PANI/Gr reaching 0.6 wt %, as shown in Figure 6c, the local impedance value significantly improved with the extension of immersion. It is important to note that the scratched area with relatively low local impedance in the PANI/Gr_0.6_-ZRP decreased in size during the immersion time extension. For the PANI/Gr_0.8_-ZRP coating, the local impedance increased explicitly after 24 h of immersion, and no shrink in size of the scratched area with relatively low local impedance was observed during immersion.

**Figure 7:**
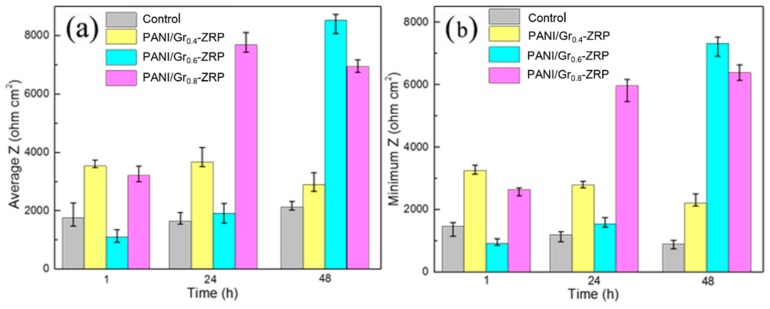
Changes in the (**a**) average impedance and (**b**) minimum impedance in the LEIS maps of Figure 6 for different immersion periods.

Figure 7 reveals the values of the average and minimum local impedance of the whole scan area. It can be observed that both the minimum values and the average values of the marked area for the PANI/Gr_0.6_-ZRP were the highest, compared to the other samples. An average impedance of 8000 Ω·cm^2^ was obtained after 48 h for the PANI/Gr_0.6_-ZRP sample, which indicated the excellent corrosion protection. In addition, the lowest impedance value of the PANI/Gr_0.6_-ZRP also sharply increased, indicating the decrease in dissolution of the exposed substrate.

Generally, for the ZRP-coated substrate, and once the coating was damaged, the zinc particles around the defect in the coating sacrificially dissolved, providing cathodic protection to the steel substrate. The result of OCP in Figure 5 demonstrates that the substrate exposed in the artificial defect was initially under the cathodic protection of the ZRP coating. However, with prolonged immersion, the sacrificial dissolution of zinc could no longer inhibit the dissolution of the substrate. The results of the LEIS in Figure 6 show the low impedance of the ZRP, which indicated the dissolution of the substrate. For the PANI/Gr-modified ZRP coatings, an increase in the local impedance was observed during the 48-h immersion, especially for the PANI/Gr_0.6_-ZRP coating. The increase in the local impedances could be attributed to the physical barrier effect of scarified zinc products, which precipitated at the defect area with the oxidization of surrounding zinc particles in the ZRP coating. It has been well documented previously in the literature that the PANI pigments in the coating on steel were found to effectively protect the substrate by forming a passive film [29,30,31]. Furthermore, it has been reported that in the presence of oxygen in the corrosive electrolyte, the PANI-EB could be converted into the PANI-ES, completing the autocatalytic cycle [32,33], which could result in the stabilization of Fe in the passive region. Thus, the flake-shape PANI in ZRP around the defects is another factor of the increase in local impedance by the formation of a protective passive film.

**Figure 8:**
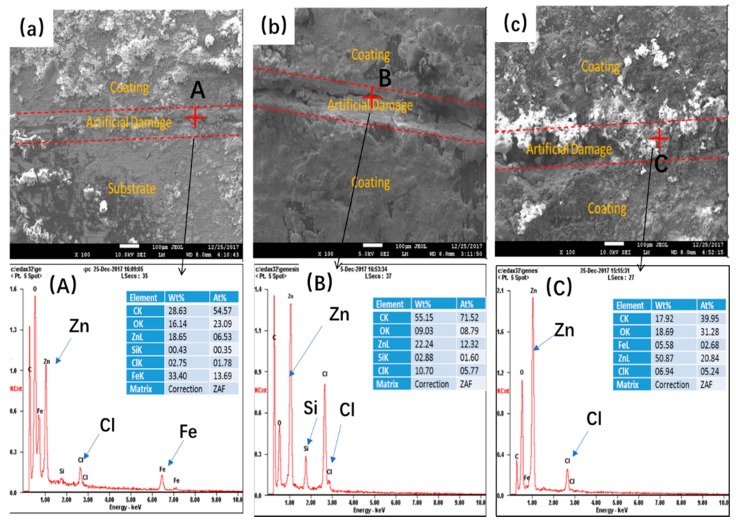
SEM images and EDS spectra for the coated samples: (**a**) PANI/Gr_0_-ZRPs, (**b**) PANI/Gr_0.6_-ZRPs, and (**c**) PANI/Gr_0.8_-ZRPs. (**A**) the EDS spectrum of the selected area marked A in (a); (**B**) the EDS spectrum of the selected area marked a in (b); (**C**) the EDS spectrum of the selected area marked C in (c).

Surface morphologies as well as the compositions of the coatings with artificial defects were investigated by SEM-EDS. Figure 8 compares the surface morphologies of the samples with different PANI contents at artificial defect zones after 30 days of immersion in 3.5% NaCl solution. It was observed that the artificial grooves were almost covered with corrosion products, composed of Zn and/or Fe corrosion products. As mentioned above, the ZRP failed to continuously support the cathodic protection with the extension of immersion, resulting in the dissolution of the substrate. From the EDS results in Figure 8, the presence of Zn elements in the control coating indicated a certain degree of cathodic protection, while the dissolution of the substrate resulted in the enrichment of Fe corrosion products. Interestingly, for the PANI/Gr_0.6_-ZRP and PANI/Gr_0.8_-ZRP samples, almost no Fe, or only a traceable amount, was detected at the defect area, which indicated that the substrate with artificial defects was effectively cathodically protected by Zn sacrificial dissolution.

**Figure 9:**
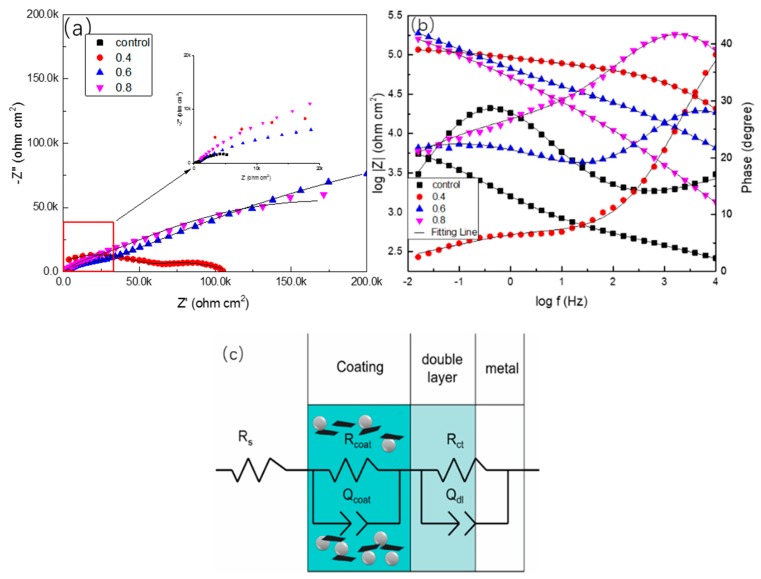
(**a**) Nyquist and (**b**) Bode plots for the samples (PANI/Gr_0_-ZRPs, PANI/Gr_0.4_-ZRPs, PANI/Gr_0.6_-ZRPs, and PANI/Gr_0.8_-ZRPs) after immersion in a 3.5 wt % NaCl solution for 25 days. (**c**) the equivalent circuit and model.

In order to investigate the barrier properties of the coatings modified with different contents of PANI/Gr, EIS was measured on the coatings. Figure 9 shows the impedance spectra for the coated samples (PANI/Gr0-ZRPs, PANI/Gr_0.4_-ZRPs, PANI/Gr_0.6_-ZRPs, and PANI/Gr_0.8_-ZRPs) in the 3.5 wt % NaCl solution after 30 days of immersion. The equivalent circuits are also presented in the spectra. It is noted that the impedance of the ZRP coatings could be significantly improved by mixing PANI/Gr, providing the barrier role. Two overlapped capacitive time constants can be distinguished at both low frequency and high frequency during the exposure to NaCl solution due to the penetration of water and oxygen into the coatings, thereby leading to the galvanic corrosion of zinc particles and the corresponding zinc corrosion products [19]. The first capacitance loop is attributed to the coating itself, and the second one is related to the reactions occurring underneath the film [34,35]. Figure 9c indicates the equivalent circuits (Rs(RctQdl)(RcoatQf)) used to simulate the EIS data. Rs refers to the solution resistance. Rct refers to the charge transfer resistance, which denotes the resistance of the electron transfer across the electron double layer from the substrate to the oxidant agent. Rcoat denotes the resistance of the coating, which arises from the kinetic resistance of the related ions or reactant agents through the coating. In order to justify the actual surface conditions of the electrode, a constant phase element (CPE, Q) was used to replace an ideal capacitor, and the impedance of Q is defined as Equation (1), as follows:

Z (jω) = (Y_0_)^−1^(jω)^−n^. (1)

In the equation, the parameters Y_0_, j, n (0 ≤ n ≤ 1), and ω (ω = 2πf, where f is the frequency) correspond to the Q constant, the imaginary unit, the Q power, and the angular frequency, respectively. It is indicated that Q is a pure capacitance when the value of n is 1. Heterogenicity of the electrode derives the value of n from the unit [36]. The impedance parameters, which were obtained using ZSimpwin software, are presented in Table 2.

For the PANI/Gr_0_-ZRPs, the film resistance (Rcoat) was only 653 Ω·cm^2^ after 30 days. This is due to the porosity of the coating which supplied the permeation path of the electrolyte. For PANI/Gr_0.4_-ZRPs, the coating resistance (Rcoat) was 7030 Ω·cm^2^ after 30 days. With the increase of PANI/Gr to 0.6 wt %, the coating resistance (Rcoat) was stable around 79,200 Ω·cm^2^. On the contrary, the coating resistance (Rcoat) of PANI/Gr_0.8_-ZRPs was lower than the coating resistance of PANI/Gr_0.6_-ZRPs, at 31,400 Ω·cm^2^. In other words, the resistance of the coatings reached a maximum value at 0.6% PANI. Another useful parameter for understanding the behavior of coatings is coating capacitance (C_coat_). Generally, the coatings with lower C_coat_ exhibit a better barrier protection against the penetration of corrosive ions [37], and water absorption causes an improvement in the coating’s dielectric constant, resulting in the increase in coating capacitance. It can be seen that the lowest C_coat_ value was obtained from PANI/Gr_0.6_-ZRP coatings after 30 days of immersion, exhibiting the best barrier properties of the PANI/Gr_0.6_-ZRP coatings. It is apparent that the highest value of coating resistance is consistent with the highest value of coating capacitance and both occurred at 0.6% PANI.

**Figure 10:**
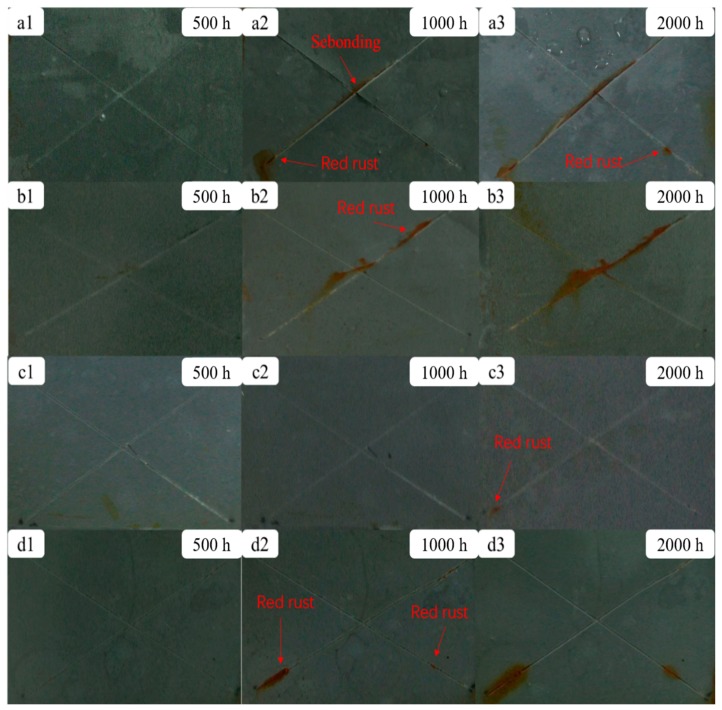
Salt spray test results of the (**a1**–**a3**) PANI/Gr_0_-ZRP, (**b1**–**b3**) PANI/Gr_0.4_-ZRP, (**c1**–**c3**) PANI/Gr_0.6_-ZRP, and (**d1**–**d3**) PANI/Gr_0.8_-ZRP samples during 2000 h exposure.

The corrosion protection properties of the ZRP coatings with different PANI/Gr contents were lastly evaluated using the salt spray test. Figure 10 shows the visual observations of the samples. Samples with X-scribes were exposed under salt spray fog for different time periods, namely, 500, 1000 and 2000 h. As shown in Figure 10, red rust formed on the scribed area of the PANI/Gr_0_-ZRPs, PANI/Gr_0.4_-ZRPs, and PANI/Gr_0.8_-ZRPs after 1000 h of salt spray testing. More corrosion products were observed at scribed regions of the PANI/Gr_0_-ZRP and PANI/Gr_0.4_-ZRP samples as the exposure time increased up to 2000 h, exhibiting poor cathodic protection performance. Compared to the control and PANI/Gr_0.4_-ZRPs, the PANI/Gr_0.8_-ZRPs showed better cathodic protection performance with the relatively low quantity of red corrosion products after 2000 h test. Interestingly, for the PANI/Gr_0.6_-ZRPs sample, no Fe corrosion products were formed after 1000 h of exposure and only a small amount of red rust appeared after 2000 h exposure. The salt spray test results revealed that PANI/Gr_0.6_-ZRPs exhibited the best cathodic protection performance for long time exposure.

**Figure 11:**
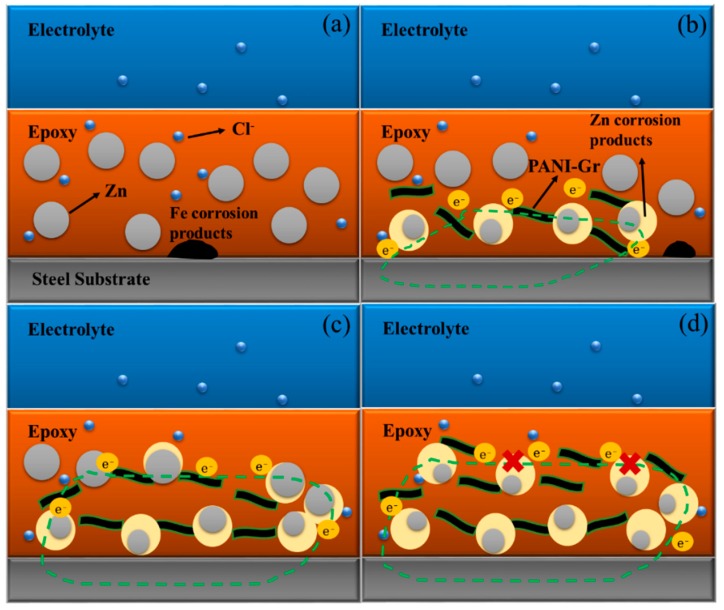
Schematic representation of (**a**) PANI/Gr_0_-ZRPs, (**b**) PANI/Gr_0.4_-ZRPs, (**c**) PANI/Gr_0.6_-ZRPs, and (**d**) PANI/Gr_0.8_-ZRPs during immersion in NaCl solution.

Mixing the PANI/Gr into the ZRP primer significantly improved the physical barrier protection due to fact that the mixture effectively restrained the permeation of caustic chloride ions to the coating and prolonged the action time of the sacrificial zinc particles. Based on the previous results, it can be seen the quantity of PANI/Gr additives in the coating has a significant effect on the corrosion behavior of the coating, and the PANI/Gr_0.6_-ZRPs coating exhibits the best anticorrosion performance. Figure 11 schematically explains the effects of PANI/Gr on the corrosion protection of ZRP primers. In the presence of 0.6 wt % PANI/Gr, the sacrificial protection is a predominant mechanism, which relates to the increase in electrical connection between the zinc particles and the steel substrate. During the immersion time, a layer of zinc corrosion products rather than iron corrosion products formed on the steel surface. For the 0.4 wt % PANI/Gr-ZRPs sample, the coating resistance was higher than the control (Figure 9), while the cathodic protection properties were not significantly enhanced (Figure 7). When the content of the PANI/Gr was further increased to 0.6 wt %, the barrier performance enhanced considerably (Figure 9), which was due to the layered structure and oxidation of zinc particles. However, if the content of Zn in the PANI is further increased (e.g., 0.8%), it may lead to too many zinc corrosion products due to the oxidation of PANI and isolate the connection between the zinc particles, resulting in a significant degradation of the cathodic protection.

## 4. Conclusions

1. Conductivity measurements indicate that the synthesized PANI/Gr shows higher conductivity compared to the PANI. OCP and LEIS results at the defect region indicate that the mixture of PANI/Gr in this investigation indeed enhances the sacrificially cathodic protection of the ZRP coating by the extension of its active sacrificial duration.
2. Higher values of coating resistance in the EIS results for PANI/Gr-modified ZRP implicate the reduction of the coating porosity, leading to the better barrier properties of the modified ZRP. The ZRP mixed with the PANI/Gr at a content of 0.6% exhibits the best synergetic enhancement of the coating physical barrier as well as enhanced zinc sacrificial cathodic protection across the range of investigated PANI/Gr mixtures.

## Figures and Tables

**Table 1:** Composition of the investigated zinc-rich paint (ZRP) coatings.

Sample	Zinc (wt %)	PANI/Gr (wt %)	Thickness (μm)
PANI/Gr_0_-ZRPs (control)	80	0	60 ± 3.4
PANI/Gr_0.4_-ZRPs	80	0.4	60 ± 2.7
PANI/Gr_0.6_-ZRPs	80	0.6	60 ± 5.3
PANI/Gr_0.8_-ZRPs	80	0.8	60 ± 4.6

**Table 2:** Optimum fit parameters of the coatings after 30 days of immersion.

Sample	R_s_ (Ω·cm^2^)	C_coat_ (Ω^−1^·cm^−2^·s^n^)	R_coat_ (Ω·cm^2^)	C_dl_ (Ω^−1^·cm^−2^·s^n^)	R_ct_ (Ω·cm^2^)
PANI/Gr_0_-ZRPs	0.253	1.36 × 10^−4^	653	3.12 × 10^−4^	7790
PANI/Gr_0.4_-ZRPs	0.575	8.38 × 10^−4^	7030	7.38 × 10^−4^	5960
PANI/Gr_0.6_-ZRPs	0.842	4.85 × 10^−5^	79200	9.64 × 10^−4^	2280
PANI/Gr_0.8_-ZRPs	0.734	1.13 × 10^−3^	31400	4.37 × 10^−4^	1820
